# Near-Infrared Spectroscopy Evaluations for the Differentiation of Carbapenem-Resistant from Susceptible Enterobacteriaceae Strains

**DOI:** 10.3390/diagnostics10100736

**Published:** 2020-09-23

**Authors:** Bushra Alharbi, Maggy Sikulu-Lord, Anton Lord, Hosam M. Zowawi, Ella Trembizki

**Affiliations:** 1 The University of Queensland Centre for Clinical Research, Faculty of Medicine, Brisbane 4029, Queensland, Australia; b.alharbi@uq.net.au (B.A.); zowawih@ksau-hs.edu.sa (H.M.Z.); 2 Faculty of Pharmacy, Taibah University, Madinah 20012, Saudi Arabia; 3 School of Public Health, The University of Queensland, Brisbane 4029, Queensland, Australia; maggy.lord@uq.edu.au; 4 Department of Immunology, QIMR Berghofer Medical Research Institute, Brisbane 4006, Queensland, Australia; Anton.Lord@qimrberghofer.edu.au; 5 Centre for Health Services Research, Faculty of Medicine, The University of Queensland, Brisbane 4029, Queensland, Australia; 6 College of Medicine, King Saud bin Abdulaziz University for Health Sciences, Riyadh 11564, Saudi Arabia; 7 King Abdullah International Medical Research Centre, Riyadh 11564, Saudi Arabia

**Keywords:** spectroscopy, near infrared, Enterobacteriaceae, carbapenem-resistant Enterobacteriaceae

## Abstract

Antimicrobial Resistance (AMR) caused by Carbapenem-Resistant Enterobacteriaceae (CRE) is a global threat. Accurate identification of these bacterial species with associated AMR is critical for their management. While highly accurate methods to detect CRE are available, they are costly, timely and require expert skills, making their application infeasible in low-resource settings. Here, we investigated the potential of Near-Infrared Spectroscopy (NIRS) for a range of applications: (i) the detection and differentiation of isolates of two pathogenic Enterobacteriaceae species, *Klebsiella pneumoniae* and *Escherichia coli*, and (ii) the differentiation of carbapenem resistant and susceptible *K. pneumoniae*. NIRS has successfully differentiated between *K. pneumoniae* and *E. coli* isolates with a predictive accuracy of 89.04% (95% CI; 88.7–89.4%). *K. pneumoniae* isolates harbouring carbapenem-resistance determinants were differentiated from susceptible *K. pneumoniae* strains with an accuracy of 85% (95% CI; 84.2–86.1%). To our knowledge, this is the largest proof of concept demonstration for the utility and feasibility of NIRS to rapidly differentiate between *K. pneumoniae* and *E. coli* as well as carbapenem-resistant *K. pneumoniae* from susceptible strains.

## 1. Introduction

Infections caused by Carbapenem-Resistant Enterobacteriaceae (CRE) are emerging as a global health concern. They are associated with difficulties in treatment and a major contributing factor to global morbidity and mortality [1]. Carbapenem-resistant pathogens are also listed as a critical priority in the World Health Organization global Priority Pathogens List [1], which primarily includes *Klebsiella pneumoniae* and *Escherichia coli*. Performing accurate, efficient and fast detection of CRE in clinical laboratories is a key factor to antimicrobial stewardship and appropriate management of patients. Access to affordable and high throughput diagnostics for surveillance of CREs is also needed, particularly in low-resource settings [2]. Various techniques are currently used in routine clinical diagnostics and surveillance to identify species and ascertain Antimicrobial Resistance (AMR). These may depend on the settings and include traditional phenotypic methods such as biochemical tests and gold standard bacterial culture methods [3].

Molecular methods, including commercial PCR-based platforms and Whole Genome Sequencing (WGS), have revolutionised clinical diagnostics and play a significant role in bacterial typing [4]. However, these methods are costly, time and labour intensive. Consequently, the practical application of these methods is not feasible in resource limited settings where disease burden is high and where syndromic-based diagnosis is the mainstay [5,6,7,8,9,10]. Ultimately, a simple, cost-effective, rapid and reproducible alternative for easy identification and characterisation of bacterial isolates and/or clinical samples should be applied.

Near-Infrared Spectroscopy (NIRS) is a technique that uses the near-infrared region of the electromagnetic spectrum (700–2500 nm) to characterise biological samples based on a reflected spectral signature. The spectral signature is collected following the interaction of biological samples with infrared light [11]. The resultant spectral signature is unique for various biological samples based on their chemical profile. NIRS is rapid and non-invasive as well as a simple technique requiring little to no sample preparation procedures and or reagents to operate [11]. NIRS is applied in multiple fields such as agriculture (e.g., to assess food quality and safety and to detect seed viability) [12,13,14], food microbiology (e.g., to assess contamination) [15], medical research (e.g., non-invasive diagnosis and pathophysiology) [16], entomology (e.g., to detect viruses in mosquitos) [17,18,19] and chemistry (e.g., measuring chemical properties of matters) [20].

There are only a handful of studies exploring NIRS to differentiate resistant from susceptible strains and one species from another [21,22,23,24,25]. The data so far are encouraging yet limited by sample size or insufficiently characterised sample banks using well-established reference methods. In addition, the variability in data analysis approaches (i.e., machine learning algorithms) and sample preparation makes it challenging to compare and assess further utility. Accordingly, in this study we aim to further close the gap and elucidate NIRS feasibility in this arena. Here, we applied NIRS on unique, well-characterised *K. pneumoniae* and *E. coli* sample banks from countries in the Middle East to (i) differentiate *K. pneumoniae* from *E. coli* and (ii) evaluate its ability to differentiate between wild-type *K. pneumoniae* from carbapenemase-producing strains.

## 2. Materials and Methods

### 2.1. Bacterial Isolates and Sample Preparation

Two bacterial species were used for this experiment and are described in detail in Table 1. *E. coli* (*N* = 40) and *K. pneumoniae* (*N* = 73). Clinical isolates were originally collected from Saudi Arabia (*E. coli n* = 2; *K. pneumoniae n* = 40), Bahrain (*K. pneumoniae n* = 1), Qatar (*E. coli n* = 4; *K. pneumoniae n* = 5), Oman (*E. coli n* = 2; *K. pneumoniae n* = 3), United Arab Emirates (*E. coli n* = 8; *K. pneumoniae n* = 6), Jordan (*E. coli n* = 19; *K. pneumoniae n* = 10), Egypt (*E. coli n* = 5; *K. pneumoniae n* = 7), Syria (*K. pneumoniae n* = 1). Bacterial species were confirmed by Matrix-Assisted Laser Desorption Ionization–Time of Flight Mass Spectrometry (MALDI–TOF MS) on a Microflex platform (Bruker Daltonics, Victoria, Australia) [26]. Initial carbapenem resistance was determined by measuring reduced susceptibility to ertapenem by disk diffusion; and DNA extracts of the isolates were PCR tested for the presence of carbapenemase determinants (*bla*_NDM_, *bla*_OXA-48_. *bla*_KPC_, *bla*_VIM_, and *bla*_IMP_ types) as previously described [27].

### 2.2. Molecular Confirmatory Analysis of Bacterial-Resistance Determinants

All *K. pneumoniae* samples (*n* = 73) were further tested by a multiplex PCR method previously described (SpeeDx Pty Ltd., Australia) [27]. Briefly, samples were screened for carbapenemase genes *bla*_KPC_, *bla*_NDM_, *bla*_OXA-48-like_, *bla*_IMP-4-like_ and *bla*_VIM_ in a single multiplex reaction. Reactions were amplified using ABI7500 real-time PCR instrument (Thermo Fisher Scientific, Waltham, MA, US) with the following cycling conditions; an initial 95 °C 2 min hold, followed by 10 touch-down cycles at 95 °C for 5 s and 61 °C (−0.5 °C per cycle) for 30 s, followed by 40 cycles at 95 °C for 5 s and 52 °C for 40 s.

### 2.3. NIR Spectroscopy

All isolates were sub-cultured twice on Mueller Hinton (MH; Becton Dickinson and company, France) plates and incubated for 12 h at 37 °C before processing with NIRS analysis. Following 24 h incubation, bacterial isolates were inoculated into 2 mL of deionised water at a cell density of 4.0 McFarland. Technical replicates (*n* = 5) of 3 μL of each bacterial suspension were placed on microscopic glass slide and were left to dry for approximately 10 min before scanning with the NIRS instrument. The dried spots were scanned with a Labspec 4*i* NIR spectrometer (Malvern Panalytical, Malvern, UK) with wavelengths ranging from 350 to 2350 nm in 1 nm increments using a fibre optic probe containing 6 illumination fibres with 600 microns surrounding a single collection fibre with 600 microns. As described in Table 1, a total of 40 biological samples of *E. coli* (with 2 replicates each; *n* = 80) and 73 biological samples of *K. pneumoniae* (with 2 replicates each; *n* = 146) were scanned by NIRS. Each spectrum is an average of 15 spectra. These were further split into 5 technical replicates for each sample, resulting in 10 data points for each biological sample (Table 1). The first 5 technical replicates of each biological sample were used as individual spectra in subsequent modelling.

### 2.4. Data Pre-Processing

The absorbance spectral data generated from the labspec 4i were converted to reflectance spectra using Equation (1). Each spectra was mean centred and normalised for variance [28,29] in R v3.5.1 [30]. Briefly, within the spectral region 700–2300 nm, all spectra were mean centred. The resulting spectra were then divided by the absolute maximum value. Outcomes (e.g., species, resistance) were coded in a binary format (0 or 1) for each classifier and predictions were generated on a continuous scale. Partial Least Squares Discriminative Analysis (PLS-DA) was then performed, using a balanced prediction cut-off of 0.5.

(1) $$A=\;\frac{\log1}{R}$$

where *A* = absorbance and *R* = reflectance.

### 2.5. Model Development and Calibration

Predictive models were developed using the PLS-DA method using the “pls” package implemented in “R” software (3.5.1) [31]. K-fold cross validation was used (*k* = 10) to validate the model. That is, data were divided into 10 groups, for each run, 9 sets of data were used to train the model with the last group used to test the accuracy. Stratified random sampling was performed (stratified according to bacterial strain) to ensure each of the 10 folds contained equal numbers of spectra from each species. Ten predictive models were developed with a different fold used as a testing set for each one. Within each fold, between 65 and 66 biological samples of *K. pneumonia* were used in the training set with the remaining 7–8 used for testing. Similarly, between 101 and 102 biological replicates of *E. coli* were used in training with the remaining 11–12 used for testing. Since each of the folds are used once and only once for testing, all 40 *E. coli* samples and 73 *K. pneumoniae* samples are reported in our testing statistics, which are aggregated across all 10 runs. The maximum number of regression factors for each model was 20. The number of factors used in each model were chosen based on the lowest number of factors required to reach the maximum accuracy within the training dataset. This process was repeated 10 times until each group had been held out once. Reported statistics are for the testing groups only. Two classification models were developed to differentiate: (1) *E. coli* from *K. pneumoniae*, and (2) *K. pneumoniae* carbapenem resistant–gene positive from *K. pneumoniae* carbapenem resistant–gene negative. Each of the models was then applied to predict the identity of samples that were not used in training the model. Accuracy, sensitivity and specificity were calculated by comparing the results against the reference methods for bacterial species confirmation and carbapenem genes detection.

## 3. Results

### 3.1. Differentiation of E. coli and K. pneumoniae

Using PLS-DA, *E. coli* and *K. pneumoniae* were differentiated with an accuracy of 89.05% (95%CI 88.7–89.4%, *p* < 0.0001) (*N* = 113). Sensitivity and specificity for differentiating the two species were 92.7% and 84.7%, respectively (Table 2). The derived models were accurate on blind data; K-fold cross validation (*k* = 10) was used. Results presented here are for the testing set.

**Figure 1:**
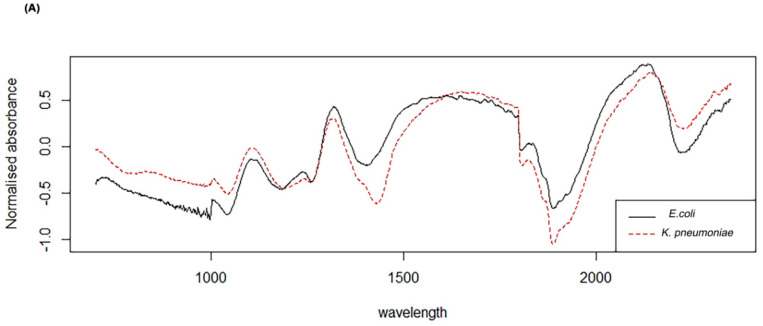

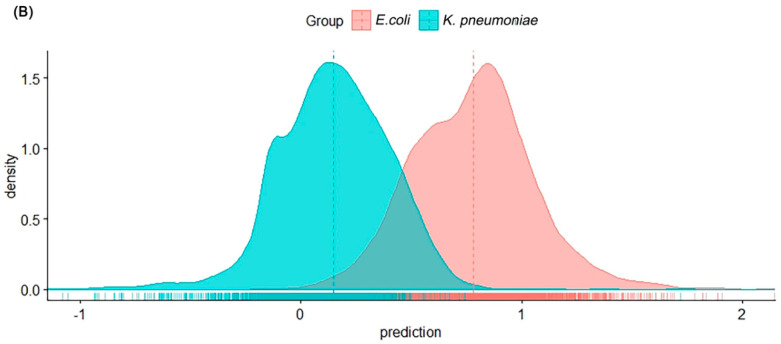
(**A**) Average Near-Infrared Spectroscopy (NIRS) spectra in the 350 to 2500 nm region from *K. pneumoniae* (red) and *E. coli* (black) and (**B**) density plot showing NIRS differentiation of *E. coli* (Pink) and *K. pneumoniae* (Blue) using test samples.

Figure 1A illustrates the normalised spectra in the region of 700–2350 nm for *E. coli* and *K*. *pneumoniae*. Accordingly, a PLS-DA was used to develop the prediction algorithm, whereby a value of 0 was assigned to *K. pneumoniae* and a value of 1 was assigned to *E. coli*. Overlapping between the two data (Pink colour was used to represent *E. coli* and Blue to represent *K*. *pneumoniae*) of the continuous interval were considered as misclassified.

### 3.2. Differentiation of Susceptible and Resistant K. pneumoniae Using NIRS

**Figure 2:**
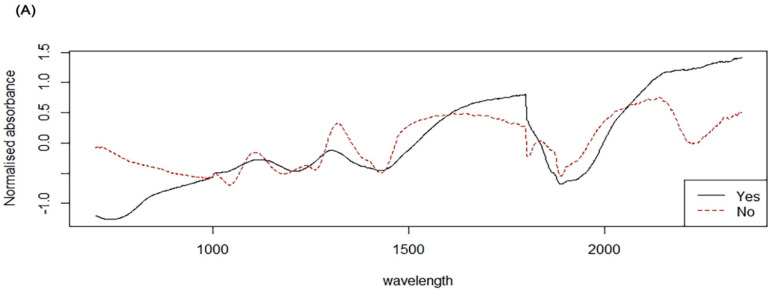

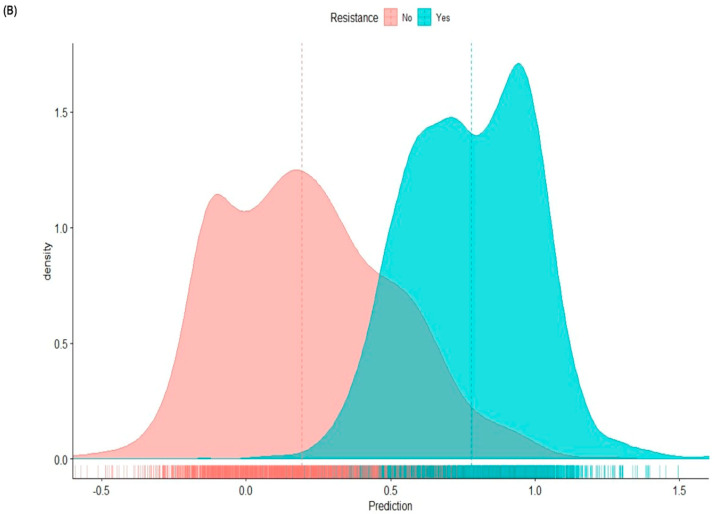
(**A**) Normalised NIR spectra in the 350–2500 nm region from susceptible *K. pneumoniae* (red line) and resistant *K. pneumoniae* (black line) Panel (**B**) Density plots showing NIRS differentiation of *K. pneumoniae* AMR-genes negative or susceptible (Pink) and AMR harbouring or resistant (Blue).

Further analysis was conducted to predict susceptibility and resistance among *K. pneumoniae* samples. The samples were previously characterised and classified as susceptible/not detected AMR mechanisms (*n* = 29) or resistant (with OXA-48 (*n* = 28), NDM (*n* = 10), and OXA-48 with NDM (*n* = 6)). In this analysis, we evaluated NIRS for its ability to differentiate susceptible *K. pneumoniae* from resistant samples regardless of the mechanism of action. The PLS model resulted in an accuracy, sensitivity and specificity of 85% (95% CI; 84.16–86.06%, *p* < 0.0001), 89% and 81%, respectively (Table 2). Figure 2A illustrates the normalised average spectra of resistant and susceptible *K. pneumoniae*. Similar to the above PLS model analysis, which was conducted as binary (1, 0) in density plot Figure 2B, a value of “0” was assigned to resistant *K. pneumoniae* and a value of “1” was assigned to susceptible *K. pneumoniae*. Overlapping between the two data of the continuous interval was considered as misclassified (Pink colour was used to represent carbapenem susceptible, while Blue colour was used to represent carbapenem resistant strains) (Figure 2B).

## 4. Discussion

The overall objective of this study was to explore the applicability and feasibility of NIRS to differentiate between *E. coli* and *K. pneumoniae*, and to differentiate between *K. pneumoniae* harbouring AMR genes from strains that are absent of AMR genes (or otherwise, wild type). Here, it was demonstrated that NIRS has the potential to differentiate these species with a predictive accuracy of 89% and can predict certain carbapenemase-encoding genes with an accuracy of 85%. Specificity and sensitivity for differentiating species (*E. coli* and *K. pneumoniae*) were 85% and 92%, respectively, and specificity and sensitivity for the AMR-gene harbouring vs. wild-type (*K. pnuemoniae*) strains were 81% and 89%, respectively.

Spectroscopy techniques to identify clinical bacteria are an emerging diagnostic approach in the medical field but are already widely applied in the food industry [32]. However, only three studies have previously explored the differentiation of bacterial species utilising NIRS that can be assessed against our study. Although sample preparation, sample size and machine learning techniques across these studies differed, predictive accuracies obtained are comparable to our results. One study utilised a miniature portable Fourier-transform NIR spectrometer (900–2600 nm) to differentiate *bla*_KPC-2_-harbouring from *bla*_KPC-2_-negative *K. pneumoniae* clinical isolates by collecting spectral signatures of bacteria DNA on aluminium-plated backing plate. Genetic Algorithm–Linear Discriminant Analysis (GA–LDA) and Successive Projection Algorithm (SPA–LDA) models were used to analyse spectral data. Predictive sensitivity using GA–LDA and SPA–LDA for *bla*_KPC_-negative was 100% and 76%, respectively, compared to the predictive sensitivity of 66% for *bla*_KPC-2_-harbouring *K. pneumoniae* using either model [25]. These data are comparable to our findings where we demonstrated that sensitivity of NIRS for predicting *blaNDM*_-type_ and *bla*_OXA-48-type_-genes harbouring *K. pneumoniae* was slightly lower (81%) than that of wild-type (92%).

A plausible limitation for the differentiation of resistant and susceptible strains in our study is the potential that the organism harbours additional resistance determinants or variations which were not previously characterised, resulting in a “false negative” call. Alternatively, the detection of a gene which is not expressed, in-turn resulting in “false positive” call. Despite the compromise on sensitivity, a way to improve accuracy is to assign a cut-off zone/value, whereby a sample will not be classified if its spectra do not fit within. This reinforces the need for further validation and testing on large well-characterised (genotype and phenotype) sample banks to best account for such variations and improve model robustness.

Kammies and colleagues investigated the use of NIRS hyperspectral imaging within the spectral region 900–2500 nm to detect and differentiate *Bacillus cereus* and two *Staphylococcus strains (aureus* and *epidermidis*). Samples were streaked onto solid Luria Broth (LB) and spectra were collected directly from the petri-dishes. Data were analysed with PLS-DA and a predictive accuracy of 90.98% (95%CI; 82–99.96%) was achieved [22]. Lastly, another group utilised Artificial Neural Network model and NIR within the range of 750 to 1350 nm to explore the differentiation of two food-borne *E. coli* strains, ATCC 25,922 (*n* = 5) and K12 (*n* = 5) grown in liquid media—a regression coefficient (R^2^) of 0.98 was achieved [23].

Here, we applied PLS-DA to differentiate the two species and to detect resistance and achieved with high predictive accuracies. It is indeed possible that other machine learning techniques would generate an improved result; however, the sample size used in our study was best suited for PLS-DA analysis. We recommend that future work with a relatively larger sample size should explore the possibility to employ other machine learning techniques for data analysis.

Finally, we demonstrated for the first time that NIRS can rapidly differentiate, with reasonable accuracy, between resistant and susceptible *K. pneumoniae* strains harbouring a range of common AMR-associated mutations. Further studies are required to assess NIRS feasibility for the identification and differentiation between and within bacterial species. Future work would include evaluating additional machine learning algorithms, increased sample size and variably, limit of detection studies, culture media comparisons to determine the effects of noise background, and finally, evaluate and develop a protocol for screening directly from clinical samples (i.e., non-culture). Importantly, a side-by-side evaluation of NIRS with Whole Genome Sequencing and phenotypical antimicrobial susceptibility data would be most advantageous for a meaningful comparable data set.

## 5. Conclusions

This proof of concept study demonstrates the potential of NIRS in microbial identification and AMR characterisation. To our knowledge, this is the largest evaluation of NIRS feasibility in differentiating *K. pneumoniae* from *E. coli*, and *K. pneumoniae* carbapenem resistant from susceptible strains. Further studies to improve model robustness and in turn improve accuracy are necessary.

## Figures and Tables

**Table 1:** Summary of *K. pneumoniae* and *E.coli* biological samples; replicate numbers and characterised molecular resistance mechanisms.

Species	Biological Samples	Replicates	Technical Replicates	Resistance Mechanism	*bla*_NDM_	*bla*_OXA-48_	*bla*_NDM_ + *bla*_OXA-48_
***K. pneumoniae***	73	2	5	47	10	29	8
***E. coli***	40	2	5 (total of 10 data points for each sample)	Not provided	-	-	-

**Table 2:** Results summary for accuracy, specificity and sensitivity for each of study analysis.

Classification Model	Accuracy%	Specificity%	Sensitivity%	*p*-Value	Total Sample Numbers (N)
*E. coli* and *K. pneumoniae* differentiation	89.04%	84.74%	92.75%	<0.0001	113
*K. pneumoniae* resistant vs. wild-type differentiation	85%	81%	89%	<0.0001	73

## References

[1] Tacconelli E., Carrara E., Savoldi A., Harbarth S., Mendelson M., Monnet D.L., Pulcini C., Kahlmeter G., Kluytmans J., Carmeli Y. (2018). Discovery, research, and development of new antibiotics: The WHO priority list of antibiotic-resistant bacteria and tuberculosis. Lancet Infect. Dis..

[2] Nordmann P., Gniadkowski M., Giske C., Poirel L., Woodford N., Miriagou V. (2012). Identification and screening of carbapenemase-producing Enterobacteriaceae. Clin. Microbiol. Infect..

[3] Richards M., Cruickshank M., Cheng A., Gandossi S., Quoyle C., Stuart R., Sutton B., Turnidge J., Bennett N., Buising K. (2017). Recommendations for the control of carbapenemase-producing Enterobacteriaceae (CPE): A guide for acute care health facilities: Australian Commission on Safety and Quality in Health Care. Infect. Dis. Health.

[4] Reuter S., Ellington M.J., Cartwright E.J., Köser C.U., Török M.E., Gouliouris T., Harris S.R., Brown N.M., Holden M.T., Quail M. (2013). Rapid bacterial whole-genome sequencing to enhance diagnostic and public health microbiology. JAMA Intern. Med..

[5] Quintelas C., Ferreira E.C., Lopes J.A., Sousa C. (2018). An overview of the evolution of infrared spectroscopy applied to bacterial typing. Biotechnol. J..

[6] Ombelet S., Ronat J.-B., Walsh T., Yansouni C.P., Cox J., Vlieghe E., Martiny D., Semret M., Vandenberg O., Jacobs J. (2018). Clinical bacteriology in low-resource settings: Today’s solutions. Lancet Infect. Dis..

[7] Poirel L., Héritier C., Tolün V., Nordmann P. (2004). Emergence of oxacillinase-mediated resistance to imipenem in *Klebsiella pneumoniae*. Antimicrob. Agents Chemother..

[8] Dortet L., Bréchard L., Cuzon G., Poirel L., Nordmann P. (2014). Strategy for rapid detection of carbapenemase-producing Enterobacteriaceae. Antimicrob. Agents Chemother..

[9] Kaase M., Szabados F., Wassill L., Gatermann S.G. (2012). Detection of carbapenemases in Enterobacteriaceae by a commercial multiplex PCR. J. Clin. Microbiol..

[10] Hirsch E.B., Tam V.H. (2010). Detection and treatment options for *Klebsiella pneumoniae* carbapenemases (KPCs): An emerging cause of multidrug-resistant infection. J. Antimicrob. Chemother..

[11] Pasquini C. (2003). Near infrared spectroscopy: Fundamentals, practical aspects and analytical applications. J. Braz. Chem. Soc..

[12] Al-Amery M., Geneve R.L., Sanches M.F., Armstrong P.R., Maghirang E.B., Lee C., Vieira R.D., Hildebrand D.F. (2018). Near-infrared spectroscopy used to predict soybean seed germination and vigour. Seed Sci. Res..

[13] Roggo Y., Duponchel L., Huvenne J.-P. (2004). Quality evaluation of sugar beet (*Beta vulgaris*) by near-infrared spectroscopy. J. Agric. Food Chem..

[14] Alander J.T., Bochko V., Martinkauppi B., Saranwong S., Mantere T. (2013). A review of optical nondestructive visual and near-infrared methods for food quality and safety. Int. J. Spectrosc..

[15] Suthiluk P., Saranwong S., Kawano S., Numthuam S., Satake T. (2008). Possibility of using near infrared spectroscopy for evaluation of bacterial contamination in shredded cabbage. Int. J. Food Sci. Technol..

[16] Sakudo A. (2016). Near-infrared spectroscopy for medical applications: Current status and future perspectives. Clin. Chim. Acta.

[17] Fernandes J.N., Dos Santos L.M., Chouin-Carneiro T., Pavan M.G., Garcia G.A., David M.R., Beier J.C., Dowell F.E., Maciel-de-Freitas R., Sikulu-Lord M.T. (2018). Rapid, noninvasive detection of Zika virus in *Aedes aegypti* mosquitoes by near-infrared spectroscopy. Sci. Adv..

[18] Sikulu M., Killeen G.F., Hugo L.E., Ryan P.A., Dowell K.M., Wirtz R.A., Moore S.J., Dowell F.E. (2010). Near-infrared spectroscopy as a complementary age grading and species identification tool for African malaria vectors. Parasites Vectors.

[19] Sikulu-Lord M.T., Maia M.F., Milali M.P., Henry M., Mkandawile G., Kho E.A., Wirtz R.A., Hugo L.E., Dowell F.E., Devine G.J. (2016). Rapid and non-destructive detection and identification of two strains of Wolbachia in *Aedes aegypti* by near-infrared spectroscopy. PLoS Negl. Trop. Dis..

[20] Kelley S.S., Rials T.G., Snell R., Groom L.H., Sluiter A. (2004). Use of near infrared spectroscopy to measure the chemical and mechanical properties of solid wood. Wood Sci. Technol..

[21] de Siqueira Oliveira F.S., Giana H.E., Silveira L. (2012). Discrimination of selected species of pathogenic bacteria using near-infrared Raman spectroscopy and principal components analysis. J. Biomed. Opt..

[22] Kammies T.-L., Manley M., Gouws P.A., Williams P.J. (2016). Differentiation of foodborne bacteria using NIR hyperspectral imaging and multivariate data analysis. Appl. Microbiol. Biotechnol..

[23] Siripatrawan U., Makino Y., Kawagoe Y., Oshita S. (2010). Near infrared spectroscopy integrated with chemometrics for rapid detection of E. coli ATCC 25922 and E. coli K12. Sen. Actuators B Chem..

[24] Marques A.S., Moraes E.P., Júnior M.A., Moura A.D., Neto V.F., Neto R.M., Lima K.M. (2015). Rapid discrimination of *Klebsiella pneumoniae* carbapenemase 2–producing and non-producing *Klebsiella pneumoniae* strains using near-infrared spectroscopy (NIRS) and multivariate analysis. Talanta.

[25] Marques A.S., Castro J.N., Costa F.J., Neto R.M., Lima K.M. (2016). Near-infrared spectroscopy and variable selection techniques to discriminate *Pseudomonas aeruginosa* strains in clinical samples. Microchem. J..

[26] Zowawi H.M., Sartor A.L., Balkhy H.H., Walsh T.R., Al Johani S.M., AlJindan R.Y., Alfaresi M., Ibrahim E., Al-Jardani A., Al-Abri S. (2014). Molecular characterization of carbapenemase-producing *Escherichia coli* and *Klebsiella pneumoniae* in the countries of the Gulf cooperation council: Dominance of OXA-48 and NDM producers. Antimicrob. Agents Chemother..

[27] Bordin A., Trembizki E., Windsor M., Wee R., Tan L.Y., Buckley C., Syrmis M., Bergh H., Cottrell K., Zowawi H.M. (2019). Evaluation of the SpeeDx Carba (beta) multiplex real-time PCR assay for detection of NDM, KPC, OXA-48-like, IMP-4-like and VIM carbapenemase genes. BMC Infect. Dis..

[28] Rinnan Å., Van Den Berg F., Engelsen S.B. (2009). Review of the most common pre-processing techniques for near-infrared spectra. TrAC Trends Anal. Chem..

[29] Pasquini C. (2018). Near infrared spectroscopy: A mature analytical technique with new perspectives—A review. Anal. Chim. Acta.

[30] R Core Team (2016). R: A Language and Environment for Statistical Computing.

[31] Mevik B.-H., Wehrens R., Liland K.H., Hiemstra P. Package ‘pls’. https://cran.rediris.es/web/packages/pls/pls.pdf.

[32] Jianxue L., Binglin Z., Denglin L., Xianfeng Z., Baocheng X., Peiyan L. (2009). Application of NIRS in the Detection of Food Microorganisms. Acad. Period. Farm Prod. Process..

[33] AlHarbi B., Lord M., Zowawi H. (2019). Rapid Identification of Bacterial Species with a Beam of Light. J. Infect. Public Health.

